# RETRACTED: Ramesh et al. Microstructure, Mechanical Characteristics, and Wear Performance of Spark Plasma Sintered TiB_2_–Si_3_N_4_ as Affected by B_4_N Doping. *Materials* 2022, *15*, 7096

**DOI:** 10.3390/ma19153322

**Published:** 2026-08-05

**Authors:** Balasubramanian Ramesh, Essmat Showman, S. A. Muhammed Abraar, Kuldeep Kumar Saxena, Mohammed Y. Tharwan, Naif Alsaadi, Sharaf Al Sofyani, Ammar H. Elsheikh

**Affiliations:** 1 Department of Mechanical Engineering, Mohamed Sathak Engineering College, Kilakarai 623806, Tamil Nadu, India; 2 Department of Mechanical Engineering, Faculty of Engineering, University of Blue Nile, Damazeen 26613, Blue Nile State, Sudan; 3 Department of Mechanical Engineering, St. Joseph’s Institute of Technology, Chennai 600119, Tamil Nadu, India; 4 Department of Mechanical Engineering, GLA University, Mathura 281406, Uttar Pradesh, India; 5 Mechanical Engineering Department, College of Engineering, Jazan University, KSA, 114 Almarefah Rd. 45142, Jazan 82874, Saudi Arabia; 6 Department of Industrial Engineering, Faculty of Engineering Rabigh Branch, King Abdulaziz University, Jeddah 21589, Saudi Arabia; 7 Industrial Engineering Department, College of Engineering, Northern Border University, Arar 91431, Saudi Arabia; 8 Department of Production Engineering and Mechanical Design, Tanta University, Tanta 31527, Egypt

The journal retracts the article titled, “Microstructure, Mechanical Characteristics, and Wear Performance of Spark Plasma Sintered TiB_2_–Si_3_N_4_ as Affected by B_4_N Doping” [1], cited above.

Following publication, concerns were brought to the attention of the Editorial Office regarding the accuracy of the data and the integrity of the images presented in this publication [1].

In accordance with the journal’s standard procedures, the Editorial Office and Editorial Board conducted an investigation that identified indications of data irregularities, as well as multiple instances of inappropriate image editing and image duplication, including the reuse of images from an earlier publication produced by a different authorship group. The authors acknowledged the presence of indicated irregularities and initiated a retraction to which the Editorial Board agreed, as they lost confidence in the reliability of the reported findings and decided to retract this publication in accordance with MDPI’s retraction policy.

This retraction was approved by the Editor-in-Chief of the journal *Materials*.

Dr. Balasubramanian Ramesh, Prof. Ammar Elsheikh, Prof. Dr. Kuldeep Kumar Saxena, Dr. Mohammed Y. Tharwan, and Dr. S. A. Muhammed Abraar have indicated their agreement with this decision. The remaining authors did not provide a comment on this decision.
